# Mutational analysis of an archaeal minichromosome maintenance protein exterior hairpin reveals critical residues for helicase activity and DNA binding

**DOI:** 10.1186/1471-2199-11-62

**Published:** 2010-08-18

**Authors:** Aaron S Brewster, Ian M Slaymaker, Samir A Afif, Xiaojiang S Chen

**Affiliations:** 1 Molecular and Computational Biology, University of Southern California, Los Angeles, CA 90089 USA

## Abstract

**Background:**

The mini-chromosome maintenance protein (MCM) complex is an essential replicative helicase for DNA replication in Archaea and Eukaryotes. While the eukaryotic complex consists of six homologous proteins (MCM2-7), the archaeon *Sulfolobus solfataricus *has only one MCM protein (ssoMCM), six subunits of which form a homohexamer. We have recently reported a 4.35Å crystal structure of the near full-length ssoMCM. The structure reveals a total of four β-hairpins per subunit, three of which are located within the main channel or side channels of the ssoMCM hexamer model generated based on the symmetry of the N-terminal *Methanothermobacter thermautotrophicus *(mtMCM) structure. The fourth β-hairpin, however, is located on the exterior of the hexamer, near the exit of the putative side channels and next to the ATP binding pocket.

**Results:**

In order to better understand this hairpin's role in DNA binding and helicase activity, we performed a detailed mutational and biochemical analysis of nine residues on this exterior β-hairpin (EXT-hp). We examined the activities of the mutants related to their helicase function, including hexamerization, ATPase, DNA binding and helicase activities. The assays showed that some of the residues on this EXT-hp play a role for DNA binding as well as for helicase activity.

**Conclusions:**

These results implicate several current theories regarding helicase activity by this critical hexameric enzyme. As the data suggest that EXT-hp is involved in DNA binding, the results reported here imply that the EXT-hp located near the exterior exit of the side channels may play a role in contacting DNA substrate in a manner that affects DNA unwinding.

## Background

DNA replication is a tightly regulated and efficient process. Central to this process is the coordinated unwinding of double stranded DNA by the AAA+ family member MCM (Minichromosome Maintenance) protein [1-3]. As the replicative helicase, MCM is required for cellular viability, and is regulated through controlled assembly at the origin in combination with ORC and Cdc6 among others, and through phosphorylation by various replication checkpoint proteins such as CDK and DDK [4-6].

In eukaryotes, MCM is composed of a heterohexamer formed from the gene products of 6 homologs (MCM2-7), all necessary for cell survival [5,7]. Archaeal MCM serves as a model system for studying MCM function as many strains only have one MCM gene whose product oligomerizes as a homohexamer or even as a double hexamer [8-10]. Several structures have been recently made available that have helped understanding of the biochemistry involved in DNA unwinding (reviewed in [11-13]. Specifically, the poorly-conserved N-terminal portion was solved in a double hexameric configuration from *Methanothermobacter thermautotrophicus *(N-mtMCM) [10], and as single hexamers from *Sulfolobus solfataricus *(N-ssoMCM) [14]. The near full length MCM monomer from *Sulfolobus solfataricus *(ssoMCM) was also recently solved [15]. Finally, the structure of an inactive MCM homolog with natural internal deletions from *Methanopyrus kandleri *(mkaMCM2) was also published [16].

The crystal structure of ssoMCM reveals an elongated fold for the monomer with two large domains. The first is a large N-terminal domain with its sequence, but not structure, poorly conserved. The second is a highly conserved C-terminal helicase domain that contains what is known as the MCM box [15]. The hexamer structures, and hexamer models of the near-full length structures, reveal a large central channel, through which DNA is postulated to be threaded.

One of the major structural features of the subunit structure of ssoMCM is the four obvious β-hairpins projecting radially away from the monomeric ssoMCM. One, located in the N-terminal domain (NT-hairpin), projects into the central channel and has been implicated in DNA binding [10,17]. The other three β-hairpins are located in the C-terminal AAA+ domain: the pre-sensor 1 hairpin (PS1-hp), the helix 2 insertion hairpin (H2I-hp), and the external hairpin (EXT-hp) [15].

Residues on the PS1-hp and H2I-hp play a role for helicase activity, are involved in DNA binding and project into or near the central channel [17,18]. The EXT-hp, however, is located on the exterior side of the hexamer, near the side channels in the C-terminal domain [15]. The unusual location of this EXT-hp raises interesting questions regarding its functional role. However, unlike the other β-hairpins, no detailed mutational and functional analysis of the EXT-hp has been performed, and its role in DNA binding, ATPase, and helicase activity is not understood. In this work we present a granular examination of this important structural feature, including a thorough examination of the hairpin's role in DNA binding, ATP hydrolysis and helicase activity.

## Results

### Mutational analysis of the exterior hairpin

**Figure 1:**
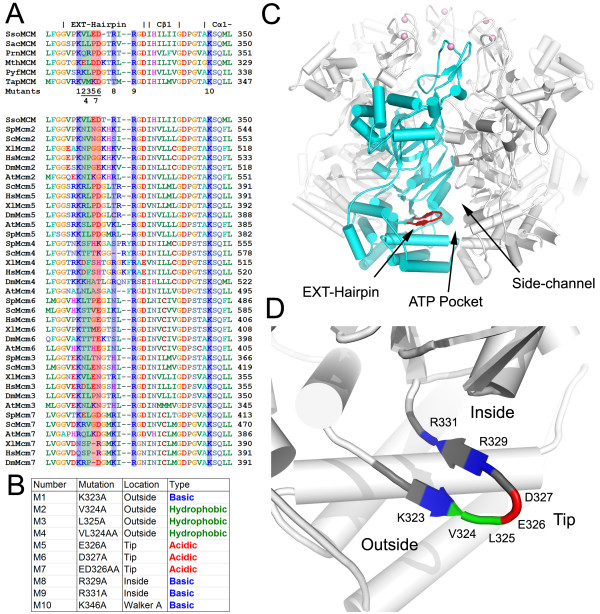
**Overview of EXT-hp mutations**. A) Sequence alignments of MCMs across archaea (top) and eukaryotes (bottom), for the short region corresponding to the EXT-hp. Sso: *Sulfolobus solfataricus*. Sac: *Sulfolobus acidocaldarius*. Prn: *Aeropyrum pernix*. Mth: *Methanothermobacter thermautotrophicus*. Pyf: *Pyrococcus furiosus*. Tap: *Thermoplasma acidophilum*. Sp: *Schizosaccharomyces pombe*. Sc: *Saccharomyces *cerevisiae. Hs: *Homo sapiens*. Xl: Xenopus *laevis*. Dm: *Drosophila melanogaster*. At: *Arabidopsis thaliana*. B) Table of mutations used for this work. C) Tilted side view of the ssoMCM hexamer model, showing the EXT-hp on the external side of the hexamer and near the side channel. A single subunit is shown in cyan, with its EXT-hp in red. The ATP binding pocket and side-channel are indicated. D) Close up view of EXT-hp. Inside and outside refer to towards the central channel and away from the central channel, respectively. The locations of the residues mutated in this study are colored on the EXT-hp according to amino acid type, as in panel B.

The EXT-hp sequence is semi-conserved in archaea and eukaryotes (Figure 1A), raising intriguing questions as to the functional roles of this hairpin next to the side channel on the exterior of the hexamer. Preliminary analysis suggested the hairpin may be involved in unwinding [15]. However, it is unclear which specific residues of the hairpin are involved in helicase function and in what manner they facilitate activity. To understand this hairpin's role in helicase function, we created a series of 10 single and double alanine mutants (numbered M1 to M10). The DNA binding, ATPase and Helicase activities were then examined for each mutant. Nine of the ten mutants are on the EXT-hp; one control mutant is on the Walker A lysine involved in ATP binding and hydrolysis (Figure 1B). The nine hairpin mutants are located in three regions, an inside β-strand that faces towards the interior side of the hexamer and side channel, a tip, and an outside β-strand that faces away from hexamer (Figures 1C, D).

Mutant M1 modifies a highly conserved lysine to alanine on the exterior side of the hairpin, close to the hairpin base. Mutants M2-M7 are located in the VLED sequence that wraps around the tip of the hairpin and consist of single or double mutations. We previously postulated that these residues could function to repel DNA, similar to the acidic pin in RuvA [15,19]. M2-M4 change the properties of the hydrophobic residues, VL. Of the two, the leucine is more conserved but neither are strongly conserved in eukaryotes or archaea. M5-M8 mutate the acidic ED region. Of note, the aspartate is highly conserved in archaea, but in eukaryotes its conservation varies by homolog. For example, in MCM5 it remains an aspartate, but in MCM3 it is consistently changed to asparagine. M8 changes a well conserved arginine in archaea that only remains consistently basic in MCM7, and mostly so in MCM2. M9, changing an absolutely conserved arginine at the base of the hairpin, had been assayed previously for ATPase and helicase activity [20], and was included as an additional control. We further assayed it for DNA binding differentials. Finally, M10, the Walker A mutant, changes the conserved lysine vital for ATP hydrolysis [9]. The Walker A motif is not conserved in the inactive MCM homolog from *Methanopyrus kandleri *[16], nor is the EXT-hp present in that homolog.

### Oligomerization as assayed by size-exclusion chromatography

**Figure 2:**
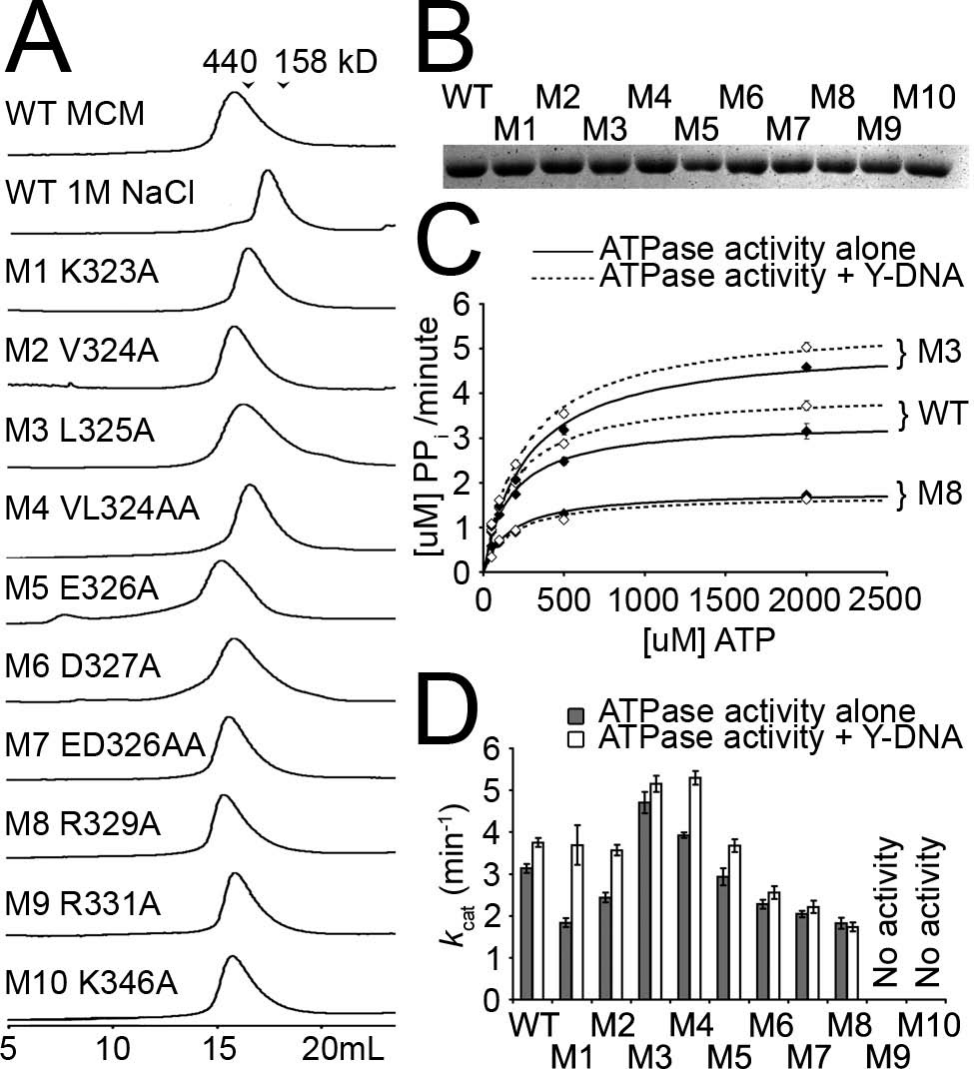
**The results of FPLC and ATPase analysis**. A) FPLC analysis of the mutations by gel filtration chromatography on a Superose-6 column. Molecular marker positions, Ferritin (440 kD) and aldolase (158 kD), are indicated by arrows. B) SDS-PAGE gel analysis of the purified mutant proteins. C) ATPase activity curves for WT, M3 and M8 in the presence and absence of Y-shaped DNA. Error bars representing standard error of the mean are present, but in most cases are too small to see. D) Summary of ATPase data in the presence (black bar) and absence (white bar) of Y-shaped DNA. Error bars are standard error from curve fitting.

We first assayed oligomeric state to determine if these mutations would affect folding or hexamerization compared with wild type. At 250 mM NaCl, wild type ssoMCM eluted on a Superose 6 size exclusion column as a hexamer. At 1 M NaCl, it eluted at a lower oligomeric state that is likely a dimer or monomer. We found previously that mutations that affect hexamerization can either cause the elution peak to shift to a lower oligomeric species, or can cause it to elute as two peaks, a hexameric peak and one at a lower oligomeric state, as if it was ran at 1 M NaCl [15]. As shown in Figure 2A, none of the 10 mutants reported here eluted as a split peak in gel filtration, and most eluted predominantly as a hexamer at 250 mM NaCl. While mutants M5 and M8 eluted slighter faster than WT and mutants M1 and M4 eluted slightly slower, their peak positions remained near the 440 kD marker (WT MCM hexamer is about 460 kD). Similar minor variations in elution profiles have been observed previously for a panel of ssoMCM mutants [20]. Thus it seems that none of the mutants have obvious defects in oligomerization, though mutants M1, M4, M5 and M8 may be slightly affected.

### ATPase activity

We next performed a series of biochemical assays of the mutants to examine their effect on ATP hydrolysis and other activities. Protein concentrations for these assays were calibrated by nano-drop and further confirmed by SDS-PAGE in Figure 2B. The first set of assays determined *k*_cat _and *k*_m _values for ATP hydrolysis via the Enzchek phosphate release assay, as described previously [15]. Activity curves for wild-type and mutants M3 and M8 are shown in Figure 2C. For each experiment, an additional test was performed to measure ATPase stimulation upon addition of Y-shaped fork DNA. WT shows a modest stimulation by the Y-shaped DNA, as noted previously. M3's ATPase activity is significantly higher than WT's. M8's ATPase activity is lower, and furthers loses ATPase stimulation upon the addition of DNA. A summary of *k*_cat _changes, together with the full data, including Km values, is given in Figure 2D and listed in Table 1.

**Table 1:** Kinetic parameters of EXT-hp mutants

	ATpase activity		ATpase + Y-DNA		ssDNA Binding		Y-DNA Binding	
	
Mutant	***k***_**cat**_, **min**^**-1**^	***k***_**m**, _**nM**	***k***_**cat**_, **min**^**-1**^	***k***_**m**, _**nM**	**k**_**d**, _**nM**	Hill	**k**_**d**, _**nM**	Hill
WT	3.1 ± 0.1	170 ± 20	3.8 ± 0.1	190 ± 20	240 ± 0.5	8.8 ± 0.5	224 ± 7.3	5.8 ± 1.3
M1	1.8 ± 0.1	140 ± 30	3.7 ± 0.5	440 ± 150	317 ± 5.8	4.4 ± 0.3	398 ± 4.0	13.2 ± 1.3
M2	2.4 ± 0.1	120 ± 20	3.6 ± 0.1	160 ± 20	209 ± 5.8	4.7 ± 0.5	230 ± 7.2	10.1 ± 3.3
M3	4.7 ± 0.3	270 ± 40	5.2 ± 0.2	260 ± 30	169 ± 1.3	5.6 ± 0.1	186 ± 6.4	7.9 ± 1.1
M4	3.9 ± 0.1	230 ± 10	5.3 ± 0.2	260 ± 30	165 ± 10.0	13.4 ± 4.3	178 ± 1.5	7.7 ± 0.5
M5	2.9 ± 0.2	60 ± 40	3.7 ± 0.2	180 ± 30	185 ± 0.3	12.1 ± 0.3	224 ± 0.9	11.6 ± 0.4
M6	2.3 ± 0.1	100 ± 20	2.6 ± 0.2	130 ± 30	283 ± 78.7	15.6 ± 20.1	400 ± 6.5	5.6 ± 0.4
M7	2.0 ± 0.1	90 ± 10	2.2 ± 0.1	90 ± 20	444 ± 1.0	10.6 ± 0.5	404 ± 2.6	8.8 ± 0.4
M8	1.8 ± 0.1	170 ± 40	1.7 ± 0.1	170 ± 30	380 ± 1.8	8.0 ± 0.3	483 ± 0.4	9.0 ± 0.03
M9	No Activity		No Activity		325 ± 8.3	6.2 ± 0.8	428 ± 2.0	12.9 ± 0.5
M10	No Activity		No Activity		267 ± 7.0	4.5 ± 0.5	339 ± 2.6	11.4 ± 2.0

All values are calculated based on monomeric MCM subunit. For each value, standard error is given. Hill: Hill cooperativity coefficient.

### DNA-binding activity

**Figure 3:**
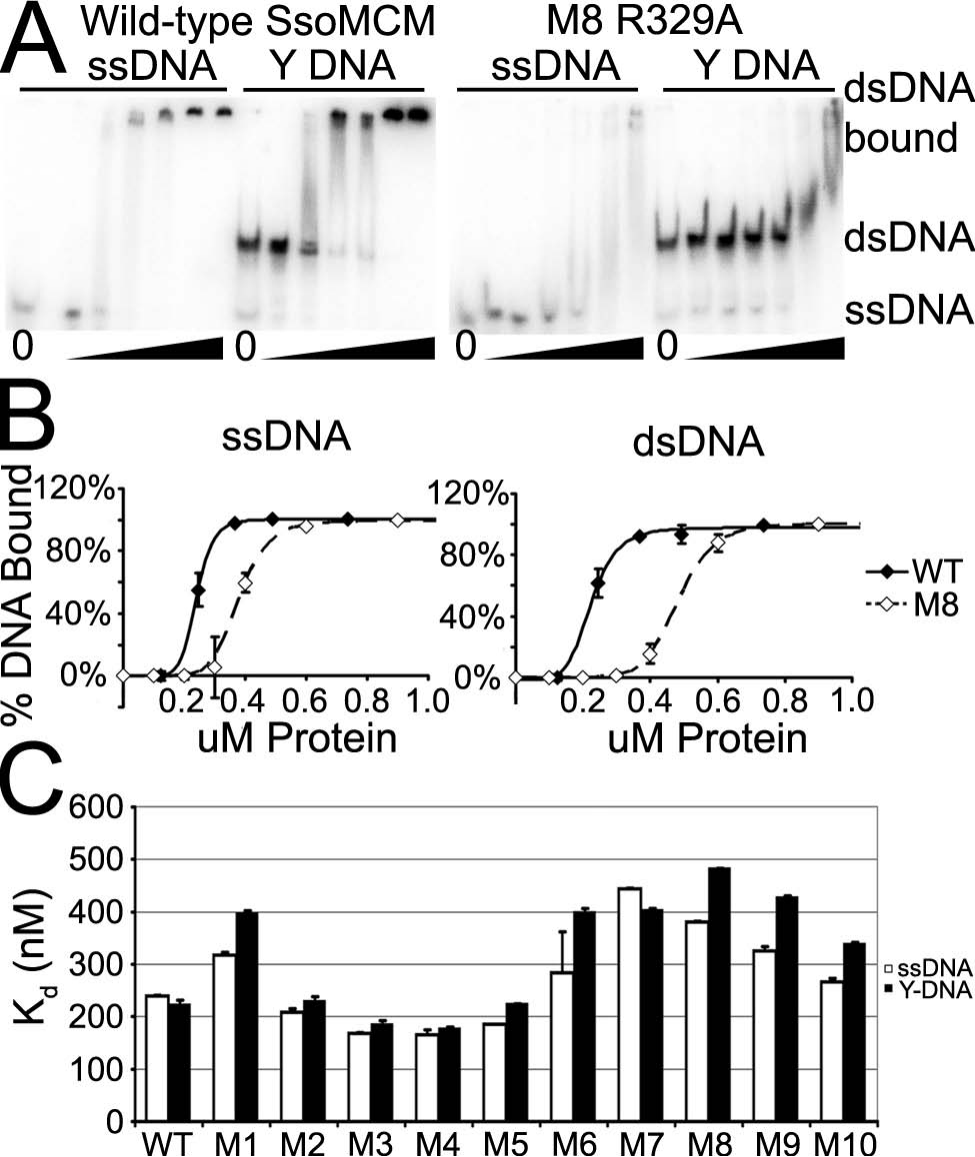
**Results of DNA binding assays**. A) Representative EMSA gels assaying for DNA binding for WT and M8. Black triangle indicates increasing protein concentration. Locations of DNA alone and DNA-protein complexes are indicated. B) The curves of DNA binding for WT and M8. Error bars represent the standard error of the mean. C) Summary of DNA binding data for all mutants. Error bars represent standard error from curve fitting.

DNA-binding activity of the mutants was determined by EMSAs using a single stranded and forked DNA substrate (Figure 3, binding constants in Table 1). Generally speaking, mutations of the hydrophobic tip (mutants M2-M4) increased DNA binding slightly. Mutations in the conserved aspartate on the tip of the hairpin (M6) showed a large decrease in dsDNA binding, and the double acidic mutant (M7) had a similar decrease in ssDNA and dsDNA binding. All of the basic mutations, including M1 on the outside of the hairpin, decreased ssDNA binding somewhat, and dsDNA binding significantly, with M8, R329A having the largest effect. These findings indicate that this hairpin is involved in DNA binding. Additionally, we see highly cooperative DNA binding in our assays. Previously, using fluorescence polarization anisotropy, we had seen Hill factors for Y-DNA binding ranging from 1.3 to 2.5 [15], suggesting cooperativity of DNA binding. Now, using EMSA assays, we see Hill factors ranging from 4 to as high as 15 for both ssDNA and Y-DNA, confirming cooperativity. The cooperative binding is likely related to the oligomerization of the six subunits of ssoMCM around the DNA. The higher Hill factors reported here could come from differences in experimental conditions; for example we have added a heating step by treating the protein-DNA mixture at 65°C for 30 minutes during sample preparation, which may allow the protein to bind DNA better.

### Helicase activity

**Figure 4:**
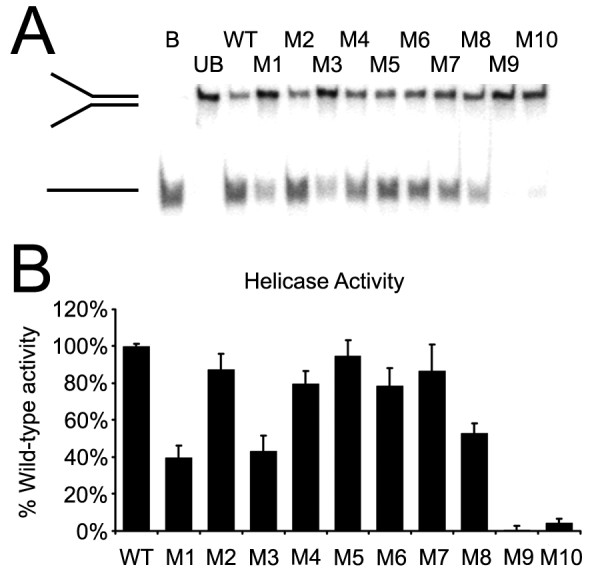
**The results of helicase assays**. A) Representative gel analysis result of helicase assay. B: boiled. UB: unboiled. DNA positions for Y-DNA and ssDNA are indicated. B) Summary of the quantified helicase activity of all mutants. Error bars represent standard error of the mean.

Helicase assays were then performed on these mutants using radio-labeled forked DNA substrates (Figure 4). As expected, the R331A and Walker A mutations, which had no detectable ATPase activity, exhibited no helicase activity, as shown previously [9,20]. Generally speaking, those mutants with lower ATPase and DNA binding activity also have lower helicase activity. However, the deficits in ATPase and DNA binding of some of the mutations did not strictly correlate with the level of deficits in helicase activity. In particular, the acidic mutations that showed significant decreases in DNA binding (M6 and M7), and decreases in ATPase activity, did not suffer significant decreases in helicase activity, at least under the assay conditions used here.

## Discussion

In this report, we conduct a systematic mutational and functional study in order to understand how this externally located β-hairpin immediately next to the exit of the side channels is involved in helicase function. We do this by assessing specific oligomerization, DNA binding, ATPase, and DNA unwinding functionality of the mutants compared with wild type MCM.

The EXT-hp contains residues with hydrophobic, acidic, and basic properties. The residues were mutated to alanine to examine the contributions of each of these residues to the various activities associated with helicase function. A summary of the data is listed in Table 2. Mutants M2-M4 contain mutations of hydrophobic residues. M2 weakens ATPase activity, but retains DNA binding and helicase activity. Its effect is minor. However, M3, located near the tip of the EXT-hp, is complex. It exhibits stronger ATPase activity than WT but its DNA induced stimulation of ATPase activity is lessened. It also has somewhat tighter DNA binding, particularly to ssDNA. Finally, its helicase activity is greatly compromised. We conclude that this mutant may slightly de-couple ATP activity from DNA unwinding. The double mutant M4 has slightly higher ATPase activity than WT but not as high as M3, and it retains DNA stimulation of the ATPase activity, DNA binding, and helicase activity.

**Table 2:** Summary of biochemical assays of the mutants

			ATPase		DNA Binding		
				
**Mutant No**.	Mutant	Oligomeric State	ATPase	Stimulation	ssDNA	Y-DNA	Helicase
WT	-	Hexamer	+++	+++	+++	+++	+++

M2	V324A	Hexamer	++	+++	+++	+++	+++
M3	L325A	Hexamer	++++	++	++++	+++	+
M4	VL324AA	Hexamer	+++	+++	++++	+++	++

M5	E326A	Hexamer	+++	+++	++++	+++	+++
M6	D327A	Hexamer	++	++	+++	+	++
M7	ED327AA	Hexamer	++	++	+	+	+++

M1	R323A	Hexamer	++	+++	++	+	+
M8	R329A	Hexamer	++	++	+	+	+
M9	R331A	Hexamer	0	0	+	+	0
M10	K346A	Hexamer	0	0	+++	++	0

Mutations are grouped by residue type (M2-M4: hydrophobic, M5-M7: acidic, M1, M8-M10: basic). +++: wild type activity level. ++++: greater than wild type (more activity or tighter binding). ++: less than wild type. +: substantially less than wild type. 0: no detectible activity. ATPase stimulation refers to the increase of ATPase activity upon addition of Y-DNA.

Mutants M5-M7 contain mutations of acidic residues (Table 2). M5 showed a slight increase in ssDNA binding, but no change from WT in all other aspects. M6, the more highly conserved of the acidic residues, revealed weaker ATPase activity, Y-DNA binding and helicase activity. It is interesting that the loss of the aspartate decreased DNA binding. The puzzling data in this set, however, is the double mutant, M7. Despite the weaker ATPase activity and DNA binding, it showed near wild-type helicase activity.

Mutants involving basic residues (M1, M8-M9) have the most profound effect in all the activities assayed. Reduced ATPase activity, DNA binding and helicase activity are observed across the board. Notably, R331 (M9), which had been assayed for ATPase and helicase activity previously [20], now is revealed to have compromised DNA binding as well.

Our single and double mutant EMSA assays from the current round of experiments reveal decreases in DNA binding on the order of 1.5 to 2 fold increases in K_d_. Mutations in the PS1-hairpin resulted in a similar change in DNA binding (2× fold increase in K_d_) [17]. Therefore, the EXT-hp appears to be involved in interactions with DNA, as shown for the three other β-hairpin structural elements in the MCM structure.

Some of the specific changes in DNA binding caused by mutations appear to be counter intuitive. For example M6 and M7, which are both mutations eliminating negative charges, have a lowered affinity for DNA. Also, the increase in binding from hydrophobic mutations is interesting. Similarly, how two basic amino acids on opposite sides of the hairpin (K323 (M1) and R329 (M8)) could impact DNA binding in similar ways is unclear. Regardless of the individual peculiarities of the mutants, there seems to be a general trend when looking at the data as a whole. With the exception of mutant M1, the functional role of the residues generally increases in importance from the base of the hairpin to the tip, with the residues located in the internal β-strand of the EXT-hairpin being the most important for ATPase activity, DNA binding and helicase activity.

The proximity of R329 (M8) (decreased DNA binding, decreased helicase activity) to the putative side-channel could implicate the hairpin in pulling ssDNA through the channel in a side channel extrusion model (see [15]). However, additional explanations are feasible. One possibility is, as proposed by Rothenberg et. al. in a DNA exclusion model [21], that one ssDNA strand may be on the exterior of the hexamer interacting with the EXT-hp while the other ssDNA is translocated through the central channel.

However, it is important to note that decreases in DNA binding were often correlated with decreases in ATPase activity. Defects in ATP binding could prevent conformational changes necessary for DNA binding. Thus these DNA binding defects could be secondary effects. Alternatively, DNA binding stimulates ATPase activity, so mutations with DNA binding phenotypes could also result in slowing the rate of ATP hydrolysis as a secondary effect. This is at least partially borne out by the fact that the *k*_m _'s for ATPase activity do not seem unduly affected by the mutations, with the exceptions of M9 and M10 (Table 1). Thus, the enzyme may be still binding ATP with wild-type affinity, but hydrolyzes ATP slower due to problems with DNA binding.

Finally, R331 (M9) is a residue of particular interest since we first examined the ssoMCM structure and compared it to known mutations [20,22]. As part of an examination of sequence alignments and structure alignments of ssoMCM with viral hexameric helicase LargeT antigen (LTag), we discovered that R331 aligns well with LTag K418 that serves as a "lysine finger" and, in combination with LTag's arginine finger, coordinates the ATP gamma phosphate in the ATP binding and hydrolysis [22-24]. K418 is vital for LTag ATPase and helicase activity [25], which mimics the phenotype we see here. Thus, the base of the EXT-hp seems directly involved in ATP hydrolysis.

## Conclusions

We have presented for the first time the systematic mutational and biochemical analysis of the residues on the EXT-hp to understand their functional roles in MCM helicase activity. The study suggests that key residues on the EXT-hp affect helicase activity through their DNA binding activity. Determining how this EXT-hp interacts with DNA through these residues and how such interactions are related to unwinding activity will require further structural and functional analysis.

## Methods

### Cloning, purification and size-exclusion analysis

WT *Sulfolobus solfataricus *MCM and mutations were cloned and purified in wash buffer (WB: 50 mM Tris-Cl (pH 8.0), 250 mM NaCl, 1 mM DTT), as described previously [15] with the following changes: prior to size-exclusion chromatography, the protein was first purified on a 6 mL Resource Q anion exchange column. Protein concentration was assayed by nano-drop and SDS-PAGE analysis. The protein was then diluted to 10 uM in helicase buffer (HB, 30 mM Tris acetate (pH 8), 75 mM NaCl, 50 mM potassium acetate, 10 mM magnesium acetate), aliquoted, and frozen at -80°C. ~0.5 mg of protein was taken separately to analyze on size-exclusion chromatography.

### Helicase assays

Helicase assays were performed exactly as described previously on radio-labeled Y-shaped DNA substrate [15]. The substrate was created from annealing two complementary strands. The sequences are: (dT)_44 _GCTCGTGCAGACGTCGAGGTGAGGACGAGCTCCTCGTGACCACG (strand Y1) and CGTGGTCACGAGGAGCTCGTCCTCACCTCGACGTCTGCACGAGC (dT)_44 _(strand Y2). Experiments were performed in triplicate.

### Electrophoretic Mobility Shift Assays

DNA binding constants were determined using electrophoretic mobility shift assays (EMSAs). ssDNA (strand Y2) or Y-shaped DNA from above was radio labeled and desalted using a Micro Bio-Spin 6 Column (BIO-RAD). Increasing amounts of protein were incubated with 1.4 nM DNA in DNA binding buffer (DB, 20 mM Tris-Cl (pH 7.5), 100 mM NaCl, 2 mM EDTA, 0.5 mM magnesium chloride) for 30 minutes at 65°C, then ran on a 5% polyacrylamide gel in 0.5× Tris/borate/EDTA buffer for 90 minutes at 100 V. DNA bands were detected by autoradiography and quantified. % DNA bound was determined vs. protein concentration and K_d _was calculated as described in [25]. Experiments were performed in duplicate.

### ATPase assays

ATPase assays were performed as described previously [15]. Experiments were performed in triplicate.

## Authors' contributions

AB designed and performed the experiments, including helicase and EMSA assays, and wrote the manuscript. IS made the clones, assisted in protein purification and performed the ATPase assays. SA assisted in protein purification and performed the size exclusion analysis. XC participated in the study's design and coordination and revised the manuscript. All authors read and approved the manuscript.
